# The importance of chorismate mutase in the biocontrol potential of *Trichoderma parareesei*

**DOI:** 10.3389/fmicb.2015.01181

**Published:** 2015-10-27

**Authors:** Esclaudys Pérez, M. Belén Rubio, Rosa E. Cardoza, Santiago Gutiérrez, Wagner Bettiol, Enrique Monte, Rosa Hermosa

**Affiliations:** ^1^Department of Microbiology and Genetics, Spanish-Portuguese Centre for Agricultural Research (CIALE), University of SalamancaSalamanca, Spain; ^2^Area of Microbiology, University School of Agricultural Engineers, University of León, Campus de PonferradaPonferrada, Spain; ^3^Embrapa EnvironmentJaguariúna, Brazil

**Keywords:** shikimate pathway, *Tparo7* gene, silencing, antifungal, tyrosol, 2-phenylethanol, salicylic acid

## Abstract

Species of *Trichoderma* exert direct biocontrol activity against soil-borne plant pathogens due to their ability to compete for nutrients and to inhibit or kill their targets through the production of antibiotics and/or hydrolytic enzymes. In addition to these abilities, *Trichoderma* spp. have beneficial effects for plants, including the stimulation of defenses and the promotion of growth. Here we study the role in biocontrol of the *T. parareesei Tparo7* gene, encoding a chorismate mutase (CM), a shikimate pathway branch point leading to the production of aromatic amino acids, which are not only essential components of protein synthesis but also the precursors of a wide range of secondary metabolites. We isolated *T. parareesei* transformants with the *Tparo7* gene silenced. Compared with the wild-type, decreased levels of *Tparo7* expression in the silenced transformants were accompanied by reduced CM activity, lower growth rates on different culture media, and reduced mycoparasitic behavior against the phytopathogenic fungi *Rhizoctonia solani, Fusarium oxysporum* and *Botrytis cinerea* in dual cultures. By contrast, higher amounts of the aromatic metabolites tyrosol, 2-phenylethanol and salicylic acid were detected in supernatants from the silenced transformants, which were able to inhibit the growth of *F. oxysporum* and *B. cinerea*. In *in vitro* plant assays, *Tparo7*-silenced transformants also showed a reduced capacity to colonize tomato roots. The effect of *Tparo7*-silencing on tomato plant responses was examined in greenhouse assays. The growth of plants colonized by the silenced transformants was reduced and the plants exhibited an increased susceptibility to *B. cinerea* in comparison with the responses observed for control plants. In addition, the plants turned yellowish and were defective in jasmonic acid- and ethylene-regulated signaling pathways which was seen by expression analysis of lipoxygenase 1 (*LOX1*), ethylene-insensitive protein 2 (*EIN2*) and pathogenesis-related protein 1 (*PR-1*) genes.

## Introduction

The use of biopesticides is an alternative for sustaining high production with low ecological impact in different agricultural production systems (Harman et al., 2010), *Trichoderma*-based products being the biofungicides available on the market most widely used (Harman et al., 2004; Lorito et al., 2010). Species of *Trichoderma* protect plants against pathogens owing to the plasticity of their genomes regarding the expression of their abilities to compete for nutrients, the inhibition or killing of plant pathogens through the production of antibiotics and/or cell wall (CW)-degrading enzymes (Druzhinina et al., 2011), the promotion of plant growth, and the induction of defenses against biotic and abiotic damage (Hermosa et al., 2012; Brotman et al., 2013).

*Trichoderma parareesei* is an efficient cellulase-producing species isolated from soil that has recently been described as a new species (Druzhinina et al., 2010). This species resembles the ancestor that originally gave rise to *T. reesei* and it exhibits the properties of an environmental opportunist (Atanasova et al., 2010). *T. parareesei* shows fast growth and produces abundant numbers of propagules on a variety of carbon sources, and is adapted to various lighting conditions. In contrast to *T. reesei*, strains of *T. parareesei* have shown biocontrol potential against fungal and oomycete plant pathogens (Atanasova et al., 2010; Rubio et al., 2014), as well as beneficial effects for plants, in terms of seedling lateral root development, and in adult plants improved defense against *Botrytis cinerea* and growth promotion under salt stress (Rubio et al., 2014).

The sequencing of *Trichoderma* genomes has revealed numerous secondary metabolite (SM) gene clusters (Mukherjee et al., 2013). The production of pyrones, peptaibols, terpenes, and polyketides is relevant in this genus (Degenkolb et al., 2006; Reino et al., 2008). Several *Trichoderma* SMs belonging to these structural families not only exhibit antagonistic activity against plant pathogens (Schirmböck et al., 1994; Rubio et al., 2009), but also have proven beneficial effects in plants (Viterbo et al., 2007; Vinale et al., 2008; Malmierca et al., 2015).

**Figure 1:**
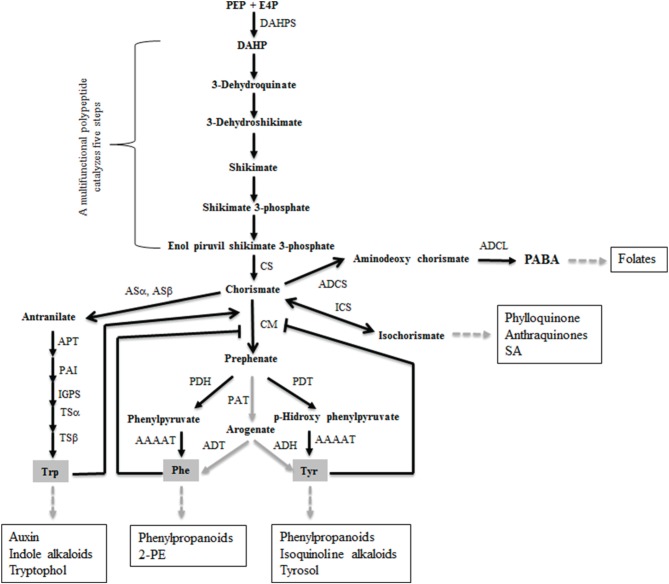
**Enzymes and metabolites comprising the shikimate pathway in *Trichoderma***. Black and gray arrows indicate the existence and absence, respectively, of genetic evidence (genes were identified in the available *T. reesei* genome and/or previously reported steps in yeast) for a given reaction. For simplicity, the AROM protein catalyzing the steps two to six is not shown. Abbreviations: PEP, phosphoenolpyruvic acid; E4P, erytrose 4-phosphate; DAHP, 3-deoxy-D-arabino-heptulosonate 7-phosphate; DAHPS, DAHP synthase; CS, chorismate synthase; CM, chorismate mutase; PDH, prephenate dehydrogenase; PDT, prephenate dehydratase; AAAAT, aromatic amino acid amidotransferase; ASα, antranilate synthase alpha subunit; ASβ, antranilate synthase beta subunit; PAT, prephenate aminotransferase; ADT, arogenate dehydratase; ADH, arogenate dehydrogenase; APT, antranilate phosphoribosyl transferase; PAI, phosphoribosylantranilate isomerase; IGPS, indole-3-glycerol phosphate synthase; TSα, tryptophan synthase alpha subunit; TSβ, tryptophan synthase beta subunit; ICS, isochorismate synthase; ADCS, 4-amino-4-deoxychorismate synthase; ADCL, adenylsuccinate lyase; 2-PE, 2-phenylethanol; SA, salicylic acid; PABA, p-amino-benzoic acid. Major metabolites derived from the shikimate pathway are marked in squared boxes.

The shikimate pathway (Figure 1) is not present in animals but it is essential in other organisms such as bacteria, fungi, or plants for the synthesis of aromatic compounds, including aromatic amino acids (AAA) tyrosine (Tyr), phenylalanine (Phe), and tryptophan (Trp) (Helmstaedt et al., 2001). Thus, it is a promising target for antimicrobial or antifungal agents and herbicides (Sasso et al., 2004). The shikimate biosynthetic route is connected to central carbon metabolism since it begins with phosphoenolpyruvate (PEP) and erythrose 4-phosphate (E-4P) (Tzin and Galili, 2010). This first step is catalyzed by the enzyme 3-deoxy-D-arabino-heptulosonate-7-phosphate (DAHP) synthase (DAHPS), which is considered to be a critical regulatory checkpoint (Bentley, 1990; Light and Anderson, 2013). The shikimate pathway includes the production of chorismate, the precursor of Tyr and Phe, through the chorismate mutase (CM) enzyme (Romero et al., 1995; Sträter et al., 1997), which also generate a wide range of SMs (Vogt, 2010). CMs can be monofunctional, where they convert chorismate to prephenate through a Claissen rearrangement, or bifunctional, where they subsequently display prephenate dehydrogenase, prephenate dehydrate, or DAHPS activities (Tohge et al., 2013). CM is an important point of regulation for maintaining the correct balance of AAA in the cell. A protein-folding structural classification separates the CMs into three classes: the AroQ prokaryotic type, the AroQ eukaryotic type, and AroH, the latter two being present in monofunctional CMs. It is known that the CM of plants and eukaryotic microorganisms such as *Saccharomyces cerevisiae* belong to the monofunctional AroQ class and that they exhibit allosteric inhibition through Tyr and/or Phe and allosteric activation by Trp (Gu et al., 1997; Krappmann et al., 1999; Tzin and Galili, 2010). It has been described that *Ustilago maydis* also secretes a monofunctional aroQ CM lacking allosteric regulation that acts as a metabolic effector in favor of this pathogen during the colonization of maize plants (Djamei et al., 2011).

In this study we describe the characterization of the *Tparo7* gene that encodes a CM in *T. parareesei*. *Tparo7*-silenced mutants were used to study the role of this primary and secondary metabolic node in *T. parareesei* biocontrol activity and its interaction with tomato plants in terms of root colonization and the induction of systemic defenses.

## Materials and methods

### Microorganisms and plants

*Escherichia coli* DH5α was used as a host for plasmid construction and propagation. This bacterial strain was grown in Luria-Bertani (LB) broth or on LB plates, supplemented with ampicillin (100 μg/ml), X-gal (40 μg/ml), and IPTG (10 μg/ml), when required.

*T. parareesei* (formerly *T. reesei*) IMI 113135 (CABI Bioscience, Egham, UK), referred to here as the T6 strain, was used as the wild-type (WT) throughout this study. T6 was used as a source of DNA to clone the *Tparo7* gene and also as a host in the transformation experiments to silence the *Tparo7* gene. All strains were propagated on potato dextrose agar (PDA, Difco Laboratories, Detroit).

*Fusarium oxysporum* CECT 2866 (Spanish Type Culture Collection, Valencia, Spain) (FO), *Rhizoctonia solani* strain19 (RS), belonging to the anastomosis group AG-2-2 IIIB, and *Botrytis cinerea* B05.10 (BC) were used as plant pathogenic microorganisms in antagonism assays. BC was used as pathogen in *in vivo* assays. These strains were stored at -80°C in 30% glycerol (FO, BC) and at 4°C in PDA plugs suspended in sterile water (RS).

Tomato seeds (*Solanum lycopersicum* “Marmande”) were sterilized in 2% sodium hypochlorite for 20 min and washed thoroughly in sterile distilled water before use.

### Culture conditions

For gene expression and CM activity studies, *T. parareesei* mycelia were obtained following a two-step liquid culture procedure (Cardoza et al., 2006). First, the strains were grown in potato dextrose broth (PDB, Difco Laboratories) at 28°C and 200 rpm for 48 h. The fungal biomass was harvested, washed and transferred to minimal medium (MM) with 2% glucose as the carbon source (Penttilä et al., 1987), and used as the control condition, or MM supplemented with 5 mM Tyr, Phe, Trp, or chorismic acid barium salt, when indicated, or PDB. After 24 h of incubation at 28°C and 200 rpm, mycelia were collected by filtration, thoroughly washed with sterile water, lyophilized, and kept at −80°C until RNA extraction.

For the determination of growth rates, fungi were inoculated at the center of MM or PDA plates and colony diameters were recorded every 24 h. To test the influence of different carbon and nitrogen sources on fungal growth, MM (with 2% glucose) or MM in which 2% glucose was replaced by 2% glycerol containing or not 38 mM ammonium sulfate or 5 mM Tyr, 5 mM Phe, and 2 mM Trp, were also used. For the determination of fungal biomass after growth in liquid culture, mycelium was collected by filtration, lyophilized and the dry weight was measured.

For the analysis of hydrolytic activities, intracellular proteins were extracted from mycelia of *T. parareesei* strains obtained from PDB containing 0.5% *R. solani* cell walls (RS-CWs) grown at 28°C and 200 rpm for 48 h. RS-CWs were obtained as previously described (Fleet and Phaff, 1974), using fungal mycelium from a PDB culture incubated at 25°C and 200 rpm for 4 days.

For metabolite quantification, *T. parareesei* supernatants were obtained from MM or PDB cultures incubated at 28°C and 200 rpm for 48 h.

To perform plant rhizosphere colonization tests, eight tomato seeds were grown in a Phytatray II box (Sigma-Aldrich) containing 100 ml of liquid MS medium (Murashige and Skoog, 1962) supplemented with 1% sucrose for 2 weeks. Each box was inoculated with 10^5^ conidial germlings ml^−1^ of *T. parareesei* strain or not (control). The collection of germlings and the maintenance of plant-fungal co-cultures were carried out as previously described (Rubio et al., 2014). After 48 h of plant-fungal interaction, the roots from eight plants per treatment were collected, washed with distilled water, frozen, lyophilized, and kept at −20°C until total DNA extraction. Three independent tomato-*T. parareesei* co-culture experiments were used for each fungal strain.

### DNA and RNA procedures

Total DNAs from fungi were extracted following the method of Raeder and Broda (Raeder and Broda, 1985), using mycelium collected from a PDB culture incubated at 28°C and 200 rpm for 2 days. DNA isolation from tomato roots was performed with the cetyltrimethylammonium bromide (CTAB) extraction method (Dellaporta et al., 1983). Total RNA was extracted using TRIZOL® reagent (Invitrogen Life Technologies, Carlsbad), following the instructions of the manufacturer.

For Southern analyses, 10 μg of genomic DNA was *Sac*I-digested, electrophoresed on a 0.7% agarose gel and transferred to a Hybond-N^+^ membrane (Amersham Biosciences AB, Uppsala, Sweden). The *phleomycin* gene was labeled using the DIG High Prime kit (Roche, Penzberg, Germany), following the manufacturer's instructions, and used as probe. Hybridization, washes and detection were carried out as previously described (Tijerino et al., 2011).

### Construction of the gene-silencing vector and transformation

Plasmid pSIL (Cardoza et al., 2006) was linearized with *Spe*I-*Bam*HI and then ligated to a 500-bp *Spe*I-*Bam*HI fragment of the *Tparo7* gene. This fragment was in “sense” relative to the orientation of the *ta* gene promoter from *T. harzianum*. The construct was then linearized with *Hin*dIII-*Xho*I and ligated to the same 500-bp *Tparo7* fragment but in an “antisense” orientation relative to the *ta* gene promoter. A 159-bp intron was introduced between both the sense and antisense fragments of the *Tparo7* gene. The resulting plasmid also contained the terminator region of the *cbh*2 gene from *T. reesei* and was designated pSIL-ARO7. In order to transform this cassette in *T. parareesei* T6, pSIL-ARO7 was digested with *Sac*I to isolate the SIL-*Tparo7* fragment, which was then ligated to the same restriction site of plasmid pJL43b1 (Gutiérrez et al., 1997), giving rise to the final construct, pJL43b1-ARO7 (7.6 kb). This latter vector was used to transform *T. parareesei* T6 by a protoplast-based method described previously (Cardoza et al., 2006). In parallel, strain T6 was also transformed with pJL43b1 to obtain empty vector transformants; one of them, Tp-TC, was included in the assays as a transformation control. Transformants were selected for phleomycin resistance.

### Real-time PCR analysis

Gene expression was analyzed by quantitative real-time PCR. cDNA was synthesized from 2 μg of RNA, which was treated with DNase RQ1 (Promega Biotech Ibérica, Alcobendas, Spain) and then used for reverse transcription with an oligo(dT) primer with the Transcriptor First Strand cDNA Synthesis kit (Takara Inc., Tokyo, Japan), following the manufacturer's protocol. Real-time PCR reactions were performed with a StepOnePlus thermocycler (Applied Biosystems, Applied Foster City) in a total volume of 10 μl, using SYBR FAST KAPA qPCR (Biosystems, Buenos Aires, Argentina) and a final primer concentration of 100 nM each. Reactions were performed with cDNA from four pooled biological replicates for each condition, with the exception of five pooled biological replicates used to analyze plant defense gene expression from *in vivo* cultures. All reactions were performed in triplicate under the following conditions: an initial denaturation step (10 min at 95°C) followed by 40 cycles of denaturation (30 s at 95°C), annealing (1 min 60°C), and extension (1 min 72°C). CT values were calculated using the Applied Biosystems software, and transcript abundance was calculated with Microsoft Excel from Ct (cycle threshold) values normalized to the *actin* gene signal. The slopes and efficiency for each primer pair, Qaro7-C/Qaro7-D, and Act-F/Act-R, were measured for dilution series of pooled cDNA samples and calculated using the Applied Biosystems software (Table S1). Relative expression levels were calculated using the 2-^ΔΔCT^ method (Livak and Schmittgen, 2001).

DNA quantification of wild-type, Tp-TC and *Tparo7*-silenced transformants from colonized tomato roots was performed by real-time PCR as previously described (Morán-Diez et al., 2009), using the primer couples Act-tomF/Act-tomR and Act-F/Act-R (Table S1) to amplify a fragment of the *actin* gene from tomato and *Trichoderma*, respectively. Mixture and real-time PCR conditions were as indicated above. Ct values were calculated and the amount of fungal DNA was estimated using standard curves. Values were normalized to the amount of tomato DNA in the samples. Each sample was tested in triplicate.

### Activity assays

Mycelia collected from liquid cultures, grown as described above, were lyophilized and homogenized in 100 mM Tris-HCl (pH 7.5) plus 10% sodium dodecyl sulfate (w/v) buffer at 800 rpm for 1 h at 4°C in a Thermomixer (Eppendorf). Protein extracts were recovered by centrifugation at 12000 × g at 4°C for 20 min and stored at −20°C. Quantitative protein determination was performed with the Bradford assay (Bradford, 1976) with bovine serum albumin (BSA) as standard protein.

CM activity was determined spectrophotometrically based on the formation of phenylpyruvate by treatment with HCl (Davidson and Hudson, 1987). The reaction mixture contained 1 mM chorismate, 0.1 mg/ml BSA, and 10 mM 2-mercaptoethanol in 50 mM Tris-HCl buffer with 1 mM EDTA, pH 8.0. For the CM activity assay, 0.4 ml of this solution was preheated for 5 min at 37°C in a water bath. After the addition of 50 μl of enzyme solution, the reaction was incubated at 37°C for 5 min. For the conversion of chorismate to phenylpyruvate, 0.4 ml of 1 M HCl was added and the mixture was further incubated at 37°C for 10 min. The samples were rendered alkaline with 1 ml of 2.5 M NaOH, and absorbance was measured at 320 nm against a blank sample without enzyme. Protease and chitinase activities were determined in colorimetric assays as previously described (Montero-Barrientos et al., 2011), measuring the hydrolysis of azocasein at 366 nm (Holwerda and Rogers, 1992), and the release of N-acetylglucosamine during the hydrolysis of chitin at 585 nm (Reissig et al., 1955), respectively. All activity measurements were performed in triplicate. Activities are expressed as μmol min^−1^ per mg of protein.

### Quantification of metabolites

One hundred ml of culture supernatant was collected by filtration through sterile filter paper and lyophilized. Then, the resulting powder was extracted with 1 ml of a H_2_O:methanol solution (9:1) and filtered through a 0.22-μm membrane. Tyrosol and 2-PE quantifications were carried out at the Mass Spectrometry Service of NUCLEUS (University of Salamanca) by capillary gas chromatography-mass spectrometry using an Agilent GC7890A gas chromatograph with a MS220 detector. A VF5ms capillary column was used (30 m, 0.25 mm 0.25 micron), with a starting temperature of 50°C kept for 5 min, followed by a ramp at 5°C per min up to 270°C, this temperature being held for 5 min.

Salicylic acid (SA) production was determined in 4 ml of culture supernatants as previously described (Leeman et al., 1996; Mercado-Blanco et al., 2001).

### *In vitro* antifungal assays

Confrontation assays (dual cultures) between *T. parareesei* strains and RS, FO, or BC plant pathogens were carried out in triplicate as previously described (Rubio et al., 2009). The dual cultures were photographed after 4 days at 28°C. BC dual cultures were also photographed after 18, 48, and 72 h at 28°C.

Growth assays on cellophane sheets were carried out in triplicate, as previously described (Rubio et al., 2009). Growth diameters were measured after 72 h for RS, 96 h for BC and after 120 h for FO. The results are expressed as the percentage of growth inhibition of each pathogen by each *T. parareesei* strain with respect to the mean colony diameters of each pathogen in control cultures.

The antifungal activity of *T. parareesei* supernatants against FO and BC was tested. A conidial suspension (2000 conidia in 5 μl) was added to the wells of sterile 96-well flat-bottomed microtiter plates along with 50 μl of water (control) or filter-sterilized (0.22-μm syringe filter; Millipore) supernatant from a 48 h-PDB culture, previously boiled for 10 min to avoid interference of enzymatic activities, or unboiled. PDB medium was added to each well up to a final volume of 150 μl. The plates were shaken briefly and placed in the dark at 28°C for 72 h. FO and BC growth were determined at 0, 24, 48, and 72 h by measuring optical density at 595 nm using a Sunrise microtiter plate reader (Tecan Ibérica, Barcelone, Spain). Each test was performed in sixtuplicate.

### *In vivo* assays

Tomato seeds were coated with a conidial suspension containing 2 × 10^8^ conidia/ml of *T. parareesei* T6, Tp-TC, or *Tparo7*-silenced transformants Tparo7-S3 and Tparo7-S4, or not (control) and were left in open Petri dishes to air-dry overnight in a laminar flow hood. One ml was used to coat 40 seeds. Plants were maintained under greenhouse conditions with a photoperiod of 16 h light: 8 h dark, a temperature between 18 and 30°C, and humidity maintained at 75% for 4 weeks. At this time, measurements of stem height and stem diameter were manually taken. Aboveground and root tissues were separated and dried until constant weight. Chlorophyll values were obtained with a Chlorophyll meter SPAD-502 Plus (Konica Minolta, Japan). The sensitivity of 4-week-old tomato plants to BC was evaluated. One leaf from each plant was inoculated on three leaflets, using 10 μl of a germination solution (20 mM glucose and 20 mM potassium phosphate) containing 2500 conidia per point. Necrotic leaf area was evaluated after three days using ImageJ free software. Ten plants were considered for each condition.

The aerial part of each tomato plant was sampled before being infected with BC and used for RNA extraction. Marker genes representative of the SA [pathogenesis-related protein 1 (*PR-1*)], jasmonic acid (JA) [lipoxygenase 1 (*LOX1*)] and ethylene (ET) [ethylene-insensitive protein 2 (*EIN2*)] signaling pathways were analyzed using real-time PCR, as described above. The primer pairs used are shown in Table S1.

### Sequence analyses

Sequences were analyzed using the DNAstar package (Lasergene, Madison). Protein sequences were aligned using the CLUSTAL X algorithm (Thompson et al., 1994). The nucleotide sequence of *Tparo7* was deposited in the GenBank database with Accession No. KT240045.

### Statistical analyses

Analysis of variance (ANOVA) was conducted with SPSS v. 19 software (SPSS Inc., Chicago) and Tukey's test was used at the 95% significance level.

## Results

### Isolation and expression of the *Tparo7* gene

A gene encoding a putative CM was identified in the available *T. reesei* genome (http://genome.jgi-psf.org/Trire2/Trire2.home.html). Based on its genomic sequence, two oligonucleotides were designed, Tparo7-F and Tparo7-R, and used to amplify a 974-bp fragment from *T. parareesei* T6 genomic DNA by PCR. This 974-bp fragment had an open reading frame (ORF) of 801 bp, two introns of 82 and 91 bp, and it was designated *Tparo7*. The ORF of *Tparo7* encodes a protein of 266 amino acids with a theoretical molecular mass of 23.94 kDa and an isoelectric point of 5.44. An analysis of the primary structure of TpARO7 revealed the conserved monofunctional aroQ eukaryotic-type CM domain from position 14–262, related to its catalytic mechanism. There was also similarity of the deduced TpARO7 sequence to cytosolic CMs from other filamentous (AROC of *Aspergillus nidulans*, 60% identity) or yeast (ARO7 of *S. cerevisiae*, 46% identity) ascomycetes, basidiomycetes (ARO7 of *U. maydis*, 40% identity), plants (*Arabidopsis thaliana*, 37–41%) or bacteria (*Chitinovibrio alkaliphilus*, 37% identity). Sequence similarity was lower with the secreted Cmu1 of *U. maydis* (12%). A secondary structure analysis of TpARO7 predicted the prevalence of the alpha-helix conformation.

The allosteric regulation effect by AAA was explored in intracellular proteins, obtained from T6 mycelia, measuring CM activity after incubation in the presence of 100 μM Tyr, Phe, or Trp, or a mixture of these three AAA at a concentration of 100 μM each. In comparison with the control condition, a 38% reduction in CM activity was recorded when 100 μM Tyr was added to the reaction mixture, whereas an activity increase of 20% was detected with 100 μM Trp. No significant changes in CM activity were observed when Phe or the AAA mixture was added.

*Tparo7* expression was investigated using real-time PCR after growing T6 for 24 h in the presence of 5 mM chorismic acid or each AAA—Tyr, Phe, or Trp. All these compounds upregulated *Tparo7* expression as compared with the transcript levels detected in 24-h MM-grown mycelia (control condition) (Figure S1). However, compared with the CM activity detected in MM, lower values were recorded from T6 cultures supplemented separately with these four compounds (Table 1).

**Table 1:** **Chorismate mutase activity of *T. parareesei* T6 intracellular protein extracts**.

	**Mean CM activity (μmol·min^−1^/mg protein) ±*SD***
MM-glucose	1.20 ± 0.09^a^
MM-Tyr	1.02 ± 0.02^ab^
MM-Phe	0.90 ± 0.10^b^
MM-Trp	1.12 ± 0.09^ab^
MM-chorismic acid	0.85 ± 0.02^b^

*Values are means of three replicates with the corresponding standard deviations. Values followed by different superscript letters are significantly different (P < 0.05). Activity was measured in mycelia from 24 h MM supplemented with 2% glucose or 5 mM Tyr, Phe, Trp, or chorismic acid barium salt cultures*.

### Isolation of *Tparo7*-silenced transformants

In order to characterize the *Tparo7* gene functionally, plasmid p43b1-ARO7 was constructed and transformed in *T. parareesei* T6. Eight out of fourteen transformants showing phleomycin resistance were checked by PCR using the primers TADIR2 and Intro-R. The presence of a 1200-bp amplification fragment was observed for seven of them. Integration of the transformation cassette was analyzed by Southern blot using *phleomycin* cDNA as a probe (Figure S2). Strains Tparo7-S2, Tparo7-S3, and Tparo7-S4, representing the three different integration patterns found among these seven transformants, and the transformation control strain Tp-TC, were chosen for further studies.

**Figure 2:**
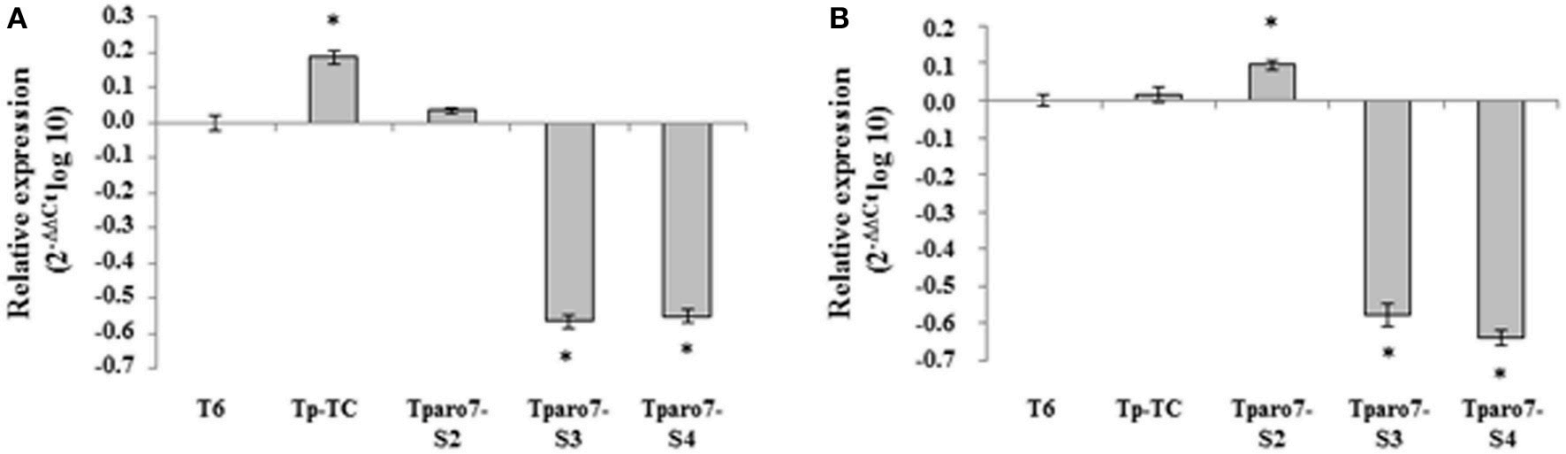
**Quantification of *Tparo7* transcripts in a control transformant (Tp-TC) and three silenced transformants (Tparo7-S2, Tparo7-S3 and Tparo7-S4) by real-time PCR**. Values correspond to relative measurements against the *Tparo7* transcript in the wild-type *T. parareesei* T6 (2^−ΔΔCt^ = 1). The experiment was carried out with mycelia grown for 48 h on PDB and transferred to MM containing 2% glucose **(A)** or PDB **(B)** for 24 h. *T. parareesei* T6 actin was used as an internal reference gene. Bars represent the standard deviations of the mean of three replicates. Asterisk (^*^) represents statistically significant differences (*P* < 0.05).

We analyzed both the expression of the *Tparo7* gene and CM activity in the selected transformants under two different culture conditions, using the expression level in strain T6 as a reference condition. The Tparo7-S3 and Tparo7-S4 transformants showed significantly lower *Tparo7* transcript levels linked to lower CM activity than those detected in T6 or Tp-TC after 24 h growth in MM and PDB (Figure 2, Table 2). Taking into account that Tparo7-S2 showed higher *Tparo7* transcript levels and CM activity than T6 or Tp-TC in PDB, the Tparo-S3 and TparoS4 strains were selected as *Tparo7*-silenced transformants for inclusion in ensuing assays. Since lower *Tparo7* expression levels were accompanied by lower CM activity, this gene must encode a CM enzyme in *T. parareesei*.

**Table 2:** **Chorismate mutase activity of *T. parareesei* T6, transformation control Tp-TC, or silenced-transformant intracellular protein extracts**.

	**Mean CM activity (μmol min^−1^/mg protein) ±*SD***	
	**MM**	**PDB**
T6	1.87 ± 0.09^b^	0.41 ± 0.01^b^
Tp-TC	2.50 ± 0.68^a^	0.33 ± 0.02^c^
Tparo7-S2	1.78 ± 0.06^b^	0.49 ± 0.03^a^
Tparo7-S3	0.70 ± 0.03^c^	0.23 ± 0.02^d^
Tparo7-S4	0.08 ± 0.05^d^	0.08 ± 0.02^e^

*Values are means of three replicates with the corresponding standard deviations. For each medium, values followed by different superscript letters are significantly different (P < 0.05). Activity was measured in mycelia from 24 h MM supplemented with 2% glucose or PDB cultures*.

CM constitutes a central node of primary metabolism. Thus, the silenced transformants displayed lower growth rates than the T6 and Tp-TC control strains in all the different culture media tested (Table 3). In addition, *Tparo7*-silenced transformants showed lower sporulation but a higher production of diffusible pigments on PDA. All strains displayed the highest colony diameters at 37°C, the optimal growth temperature for *T. parareesei*, and significantly lower sizes were observed in the silenced mutants in comparison with those from T6 and Tp-TC (data not shown).

**Table 3:** **Growth rates of *T. parareesei* strains on different culture media**.

	**PDA**	**MM-glucose**	**MM-glycerol**	MM-glycerol-AAA^*^
T6	7.0 ± 0.1^a^	4.2 ± 0.0^a^	3.8 ± 0.1^a^	3.0 ± 0.1^a^
Tp-TC	7.0 ± 0.1^a^	4.1 ± 0.1^a^	3.9 ± 0.1^a^	3.2 ± 0.2^a^
Tparo7-S3	4.2 ± 0.0^c^	1.9 ± 0.1^b^	1.5 ± 0.2^c^	0.7 ± 0.2^b^
Tparo7-S4	5.0 ± 0.3^b^	2.0 ± 0.1^b^	1.9 ± 0.0^b^	0.9 ± 0.1^b^

* *The ammonium sulfate of the MM was replaced by a mix containing 5 mM Tyr, 5 mM Phe, and 2 mM Trp*.

*Values are means of three replicates with the corresponding standard deviations. For each medium, values followed by different superscript letters are significantly different (P < 0.05). Colony diameters (cm per 24 h) of wild-type (T6), transformation control (Tp-TC) and Tparo7-silenced transformants (Tparo7-S3 and Tparo7-S4) were measured on agar plates containing PDA or MM supplemented with 2% glucose or 2% glycerol*.

### Antifungal activity

**Figure 3:**
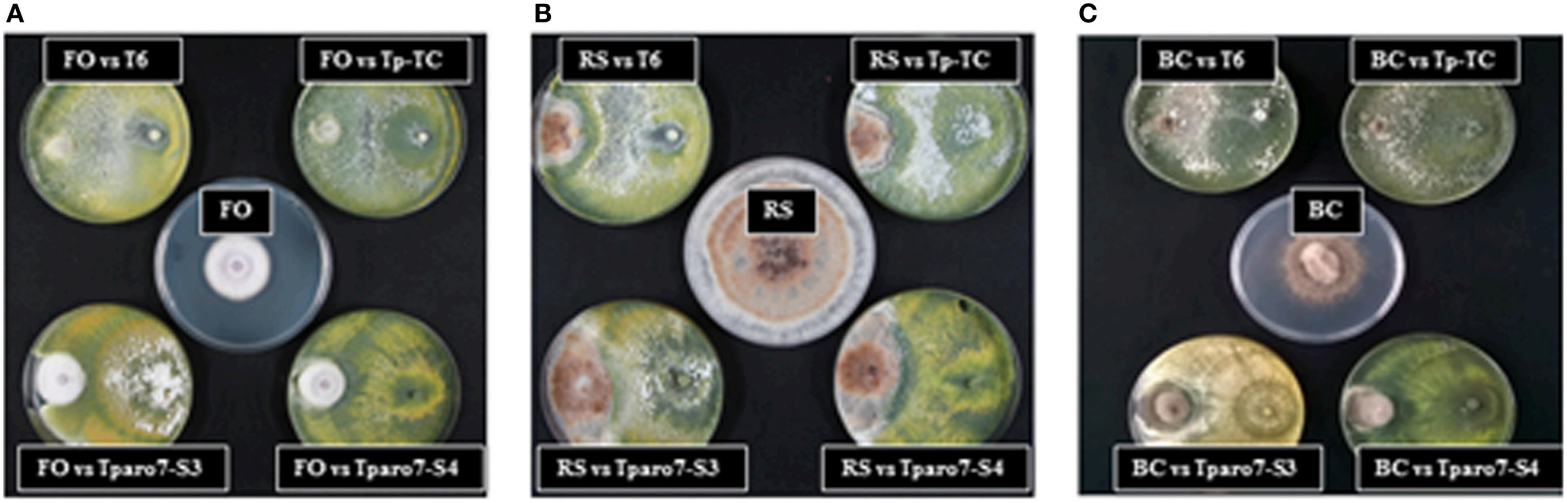
**Dual cultures of strains T6, the silenced transformants Tparo7-S3 and Tparo7-S4, and the control transformant Tp-TC of *T. parareesei* and the pathogens *F. oxysporum* (FO) (A), *R. solani* (RS) (B), and *B. cinerea* (BC) (C) on PDA medium**. Plates in the center correspond to the growth of each pathogen without *Trichoderma* strains. Plates were incubated at 28°C for 4 days.

The influence of *Tparo7* silencing in the antifungal activity of *T. parareesei* was evaluated using different *in vitro* assays with the phytopathogens FO, BC and/or RS as targets. A lower mycoparasitic behavior was observed for *Tparo7*-silenced transformants than for T6 or Tp-TC in dual confrontation experiments performed between the *T. parareesei* strains and the three targets (Figure 3 and Figure S3). After 4 days of incubation, T6 and Tp-TC were able to overgrow the colonies of FO or BC, whereas Tparo7-S3 and Tparo7-S4 only surrounded them. This absence of FO and BC overgrowth capacity by the silenced transformants persisted even at 10 days of incubation (data not shown).

The antagonistic potential of *T. parareesei* extracellular compounds against these pathogens was evaluated using a membrane assay. After removal of the cellophane sheet containing the *T. parareesei* mycelium, the effect of the total compounds secreted by antagonistic strains on the growth of pathogens was determined. The FO, RS, and BC growth inhibition percentages calculated for the four *T. parareesei* strains are summarized in Table 4. The compounds secreted by T6 or Tp-TC displayed a more marked inhibition of FO and RS than those secreted by *Tparo7*-silenced mutants (*P* < 0.05). However, the highest and the lowest inhibition values of BC were respectively obtained from plates where strains Tparo7-S3 and Tparo-S4 had been previously grown.

**Table 4:** **Colony growth inhibition (%) of *F. oxyxporum, R. solani* and *B. cinerea* by *T. parareesei* strains grown on cellophane membranes**.

	***F. oxyxporum***	***R. solani***	***B. cinerea***
T6	28.3 ± 0.4^a^	24.0 ± 0.0^a^	22.7 ± 1.7^c^
Tp-TC	28.1 ± 0.4^a^	24.0 ± 0.2^a^	26.2 ± 0.8^b^
Tparo7-S3	26.2 ± 0.3^b^	11.0 ± 0.1^b^	29.8 ± 0.2^a^
Tparo7-S4	16.0 ± 0.1^c^	2.0 ± 0.1^c^	8.5 ± 0.5^d^

*Values are means of three replicates with the corresponding standard deviations. For each column, values followed by different superscript letters are significantly different (P < 0.05). Strains were wild-type (T6), transformantion control (Tp-TC), and Tparo7-silenced transformants (Tparo7-S3 or Tparo7-S4)*.

To analyze the effect of *Tparo7* silencing in the production of CW hydrolytic enzymes by *T. parareesei* strains, we measured protease and chitinase activities on intracellular proteins from 48-h PDB plus 0.5% RS-CWs-grown mycelium (Table 5). Lower values of protease activity were observed for the silenced transformants Tparo7-S3 and Tparo7-S4 than for strains T6 or Tp-TC, although such differences were only significant for Tparo7-S4 (*P* < 0.05). However, strain Tparo7-S3 displayed significantly higher chitinase activity than any other strain.

**Table 5:** **Protease and chitinase activities measured in *T. parareesei* strain intracellular protein extracts obtained from 48 h liquid cultures in PDB supplemented with 0.5% *R. solani* cell walls**.

	**Mean activity (μmol·min^−1^/mg protein) ±*SD***	
	**Protease**	**Chitinase**
T6	56.83 ± 0.67^a^	0.78 ± 0.09^b^
Tp-TC	57.84 ± 3.80^a^	0.64 ± 0.03^c^
Tparo7-S3	48.07 ± 5.83^ab^	1.93 ± 0.25^a^
Tparo7-S4	24.65 ± 0.88^b^	0.90 ± 0.07^b^

*Values are means of three replicates with the corresponding standard deviations. For each activity, values followed by different superscript letters are significantly different (P < 0.05)*.

**Figure 4:**
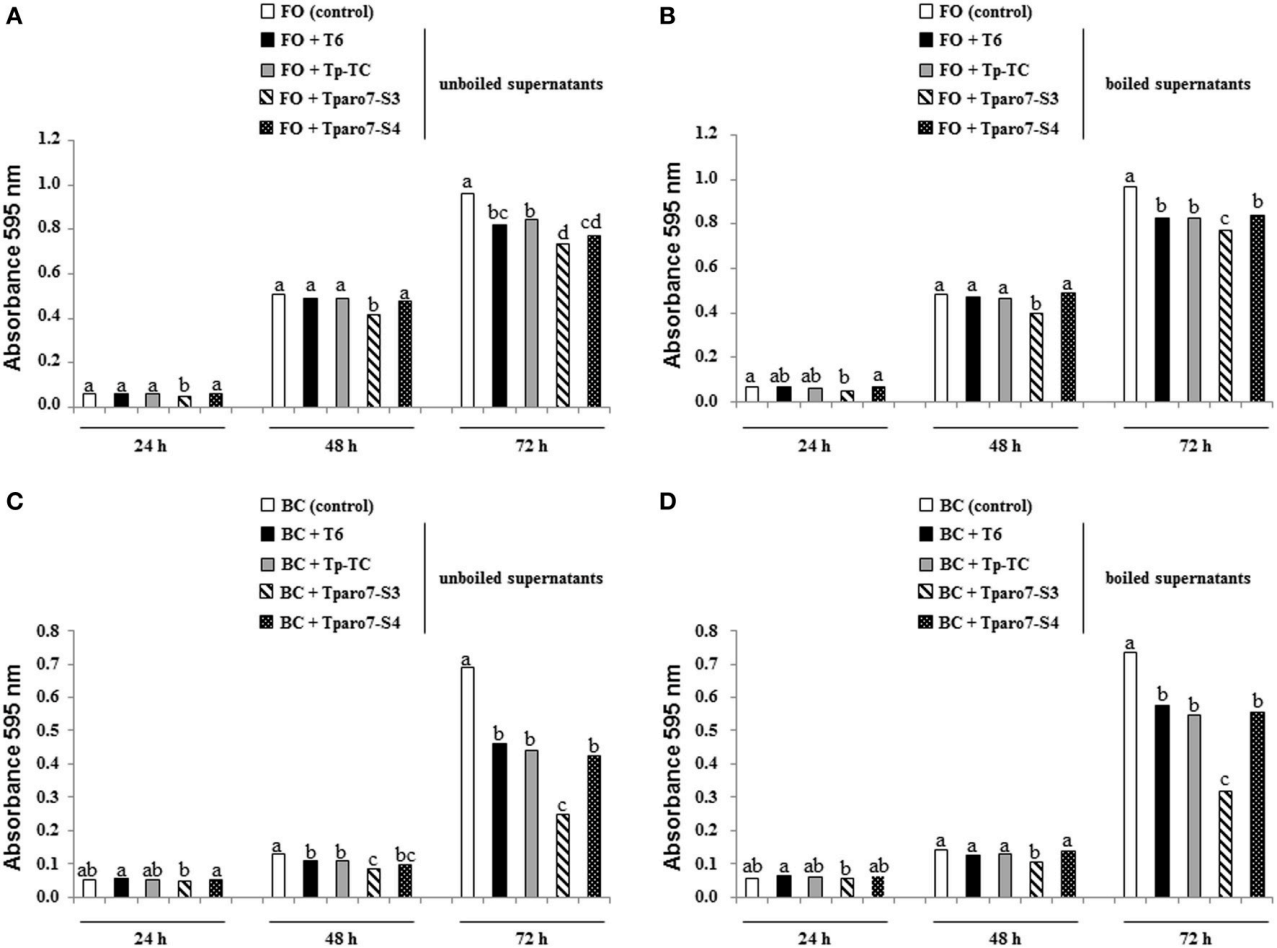
**Effect of *T. parareesei* supernatants in the growth of *F. oxysporum* (FO) (A,B) and *B. cinerea* (BC) (C,D)**. Tests were carried out without (control) or with 50 μl of filter-sterilized supernatant from 48 h-PDB cultures of strains T6, Tp-TC, Tparo7-S3 and Tparo7-S4, previously boiled for 10 min **(B,D)** or not **(A,C)**. Fungal growth was determined after 28°C incubation at 24, 48, and 72 h by measuring absorbance at 595 nm using a microtiter plate reader. Values are means of six replicates. Bars corresponding to every pathogen, supernatant treatment and incubation time marked with different letters are significantly different (*P* < 0.05).

The antifungal activity of *T. parareesei* supernatants was evaluated against FO and BC on 96-well E plates. In these tests, the hyphal growth from conidia of the two target fungi was registered at 24, 48, and 72 h, and unboiled and boiled supernatants were considered to determine the antifungal activity of SMs plus secreted enzymes and SMs, respectively (Figure 4). The absorbance values recorded at 0 h did not show significant differences among the five conditions considered in these tests (data not shown). The supernatants from the different *T. parareesei* strains had significant antifungal activity against FO and BC at 72 h. Moreover, Tparo7-S3 supernatants, unboiled and boiled, showed significant higher inhibitory effect against the two pathogens compared to that of T6 or Tp-TC supernatants at 48 and 72 h. In a wide sense, *T. parareesei* supernatants were more effective against BC and SMs looked to be more relevant for their antifungal activity against both pathogens.

### Metabolite production

To analyze the effect of *Tparo7* silencing on shikimate pathway metabolite production, tyrosol, 2-PE and SA were determined in MM and PDB supernatants from 48-h-old cultures (Table 6). These metabolites were not detected in MM for any strain. Higher amounts of tyrosol, 2-PE and SA were measured in PDB supernatants of *Tparo7*-silenced transformants than those observed for T6 or Tp-TC. Similar results were obtained in two independent biological experiments.

**Table 6:** **Concentration of tyrosol, 2-PE and SA in *T. parareesei* T6, Tp-TC, Tparo7-S3, and Tparo7-S4 strain supernatants obtained from 48 h PDB cultures**.

	**Tyrosol**		**2-PE**		**SA**	
T6	18.98	19.04	9.49	9.52	0.09	0.12
Tp-TC	20.33	21.01	11.96	12.36	0.08	0.08
Tparo7-S3	33.27	33.60	27.40	27.66	0.73	0.59
Tparo7-S4	32.13	27.21	28.11	23.81	0.15	0.21

*Tyrosol and 2-PE are expressed as mg per g of mycelial dry weight, and SA is expressed as μg per g of mycelial dry weight. Values correspond to two independent biological replicates*.

In order to analyze the biocontrol potential of tyrosol, 2-PE and SA, putatively formed in the metabolic AAA pathway, we tested the effect of these compounds on the mycelial growth of RS, FO, and BC. The growth inhibition percentages of FO, RS and BC by 5, 10, and 30 mM of commercial tyrosol, 2-PE, or SA, concentrations no biologically relevant, are shown in Table S2. In general, growth inhibition values were directly proportional to the concentration of tyrosol, 2-PE, or SA in the culture medium. The growth of these plant pathogens was completely inhibited by 30 mM 2-PE or SA, and inhibition percentages of about 55% were obtained with 30 mM tyrosol.

### Tomato rhizosphere colonization

The role of *Tparo7* in plant rhizosphere colonization was evaluated on tomato roots by quantitative real-time PCR using T6 and Tp-TC as controls and the two *Tparo7*-silenced transformants. As shown in Table 7, the amount of Tparo7-S3 and Tparo7-S4 DNA obtained from tomato roots was significantly lower than those of strains T6 and Tp-TC (*P* < 0.05).

**Table 7:** Colonization of tomato roots by *T. parareesei* wild-type (T6), control transformant (Tp-TC), and *Tparo7*-silenced transformants (Tparo7-S3 or Tparo7-S4)^*^.

**Strain**	**Tomato actin**			***Trichoderma*** **actin**			Ratio^****^
	**Ct**	**SD**	Qty^**^	**Ct**	**SD**	Qty^***^	
T6	20.82	0.06	144.52	16.87	0.02	24.58	0.17 ± 0.01^a^
Tp-TC	20.97	0.02	127.65	17.01	0.18	22.07	0.17 ± 0.03^a^
Tparo7-S3	21.04	0.10	121.25	18.43	0.31	7.74	0.06 ± 0.02^b^
Tparo7-S4	21.78	0.09	67.43	18.93	0.03	5.38	0.08 ± 0.01^b^

* *Fungal DNA present on the tomato roots 48 h after the inoculation was quantified by real-time PCR. Three replicates were made of each sample. Ct, threshold cycle and SD, standard deviation*.

** *Quantity of tomato DNA (ng) referred to tomato actin gene*.

*** *Quantity of Trichoderma DNA (ng) referred to Trichoderma actin gene*.

**** *Proportion of fungal DNA vs. plant DNA. Values are the means of the three replicates with the corresponding standard deviations and values followed by different superscript letters are significantly different (P < 0.05)*.

### Biocontrol in tomato

Four-week-old tomato plants previously seed-coated with an aqueous solution (control) or treated with conidia of T6, Tp-TC, Tparo7-S3, or Tparo7-S4 were evaluated in this assay. The *in vivo* assay results revealed that there were significant differences in stem length, aboveground and root dry weights, chlorophyll SPAD index and the leaf area of 4-week-old tomato plants between T6 or Tp-TC and *Tparo7*-silenced strain treatments (Table 8). The lowest size, dry weight and chlorophyll value were observed in tomato plants previously seed-coated with *Tparo7*-silenced transformants. These data indicated that *Tparo7* gene silencing negatively affects tomato plants. The results obtained in BC-infected tomato assays with strains T6, Tp-TC, Tparo7-S3 and Tparo7-S4 showed that *Tparo7* silencing also affected the biocontrol activity of *T. parareesei* (Table 8, Figure S4). The highest necrotic leaf area was observed in *Tparo7*-silenced transformant treatments and no statistically differences were observed among the necrotic lesion sizes observed for the T6 and Tp-TC treatments.

**Table 8:** Effect of tomato seed treatment with *T. parareesei* T6, Tp-TC, Tparo7-S3 or Tparo7-S4 strains or not (control) on tomato plant growth (4-week-old plants) and necrotic leaf area caused by B. cinerea^*^.

**Treatment**	**Control**	**T6**	**Tp-TC**	**Tparo7-S3**	**Tparo7-S4**
Stem diameter (mm)	5.31^a^	5.24^a^	5.21^a^	3.81^b^	4.12^b^
Stem height (cm)	34.40^a^	33.80^a^	36.20^a^	19.60^b^	19.50^b^
Aboveground dry weight (g)	1.81^a^	1.40^a^	1.68^a^	0.49^b^	0.53^b^
Root dry weight (g)	0.28^a^	0.27^a^	0.29^a^	0.15^b^	0.16^b^
Chlorophyll (SPAD index)	25.70^a^	24.60^a^	25.40^a^	18.70^b^	20.30^b^
Foliar area (cm^2^)	8.42^a^	7.09^a^	7.53^a^	4.01^b^	5.53^b^
Necrotic foliar area (%)^*^	1.61^b^	1.59^b^	1.52^b^	21.68^a^	16.85^a^

*Ten plants were considered for each condition. Foliar area and necrotic foliar area were evaluated using ImageJ software*.

* *One leaf from each plant was inoculated on three leaflets using 10 μl containing 2500 B. cinerea conidia/point and the necrotic leaf area was evaluated after 3 days. In each line, means followed by different superscript letters are significantly different (P < 0.05)*.

**Figure 5:**
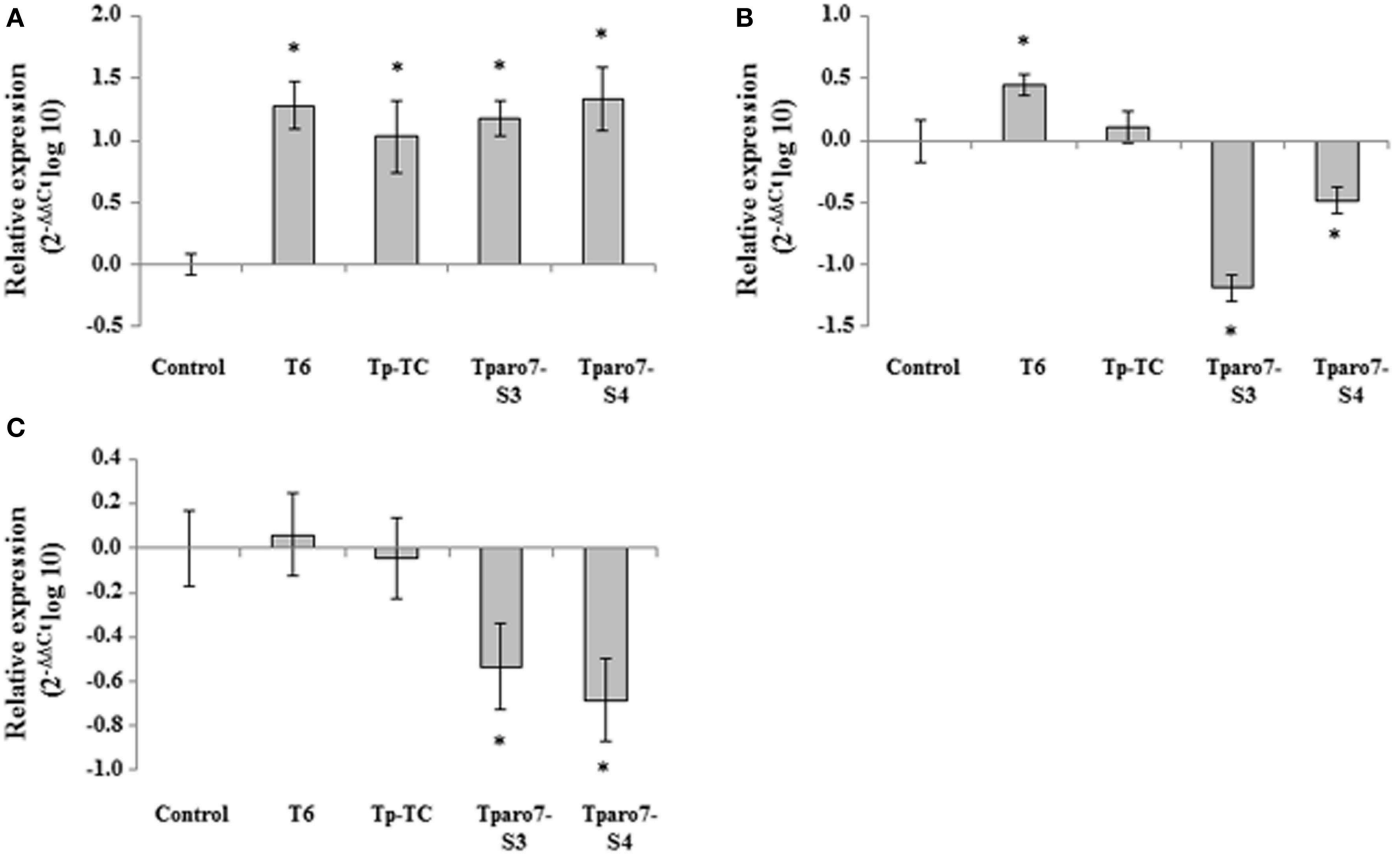
**Relative expression of the *PR*-1 (A), *LOX1* (B), and *EIN*2 (C) genes in 4-week-old tomato leaves from seeds coated with a conidial suspension of *T*. *parareesei* T6, Tp-TC, Tparo7-S3, and Tparo7-S4**. Values correspond to relative measurements against the transcripts in tomato leaves from untreated seeds (2^−ΔΔCt^ = 1). Tomato *actin* was used as an internal reference gene. Bars represent the standard deviations of the mean of three replicates. Asterisk (^*^) represents statistically significant differences (*P* < 0.05).

To test whether the tomato response to the different *T. parareesei* treatments involved the differential activation of systemic defense-related genes, we analyzed markers of the SA (*PR-1*), JA (*LOX1*), and ET (*EIN2*) pathways using real-time PCR in tomato leaves from 4-week-old plants. Marker gene expression in tomato plants from *in vivo* assays is shown in Figure 5. In comparison with the control, *T. parareesei* seed-coated tomato plants showed significantly increased expression levels of *PR-1*, whereas no differences were detected among strains. This result shows that SA pathway was induced at 4 weeks after *T. parareesei* treatment, which indicates a long-term systemic acquired resistance response elicited by *T. parareesei*. At the same time, *EIN2* and *LOX1* were downregulated in tomato seedlings from seeds treated with the *Tparo7*-silenced transformants and no statistically significant differences were observed among their expression levels in control plants and Tp-TC seed-coated plants.

## Discussion

Because the use of biofertilizers and biopesticides is an alternative for sustaining high production with low ecological impact, the development of research addressing biological control is currently expanding exponentially. Regarding the biocontrol of plant diseases, *Trichoderma*-based products are the most important biofungicides (Lorito et al., 2010), because selected *Trichoderma* strains have beneficial effects on plants, which can be explained in terms of plant growth promotion and the biological control of plant pathogens (Hermosa et al., 2012). Although *T. parareesei* T6 was able to achieve its best growth at 37°C and this temperature is closely related to the optimal growth temperature range of human pathogens, this species has not been neither isolated nor related to human disease and the continuous use of this strain in the laboratory has not resulted hazardous. In any case, risk assessment studies are needed before considering any *in vivo* application of this species in commercial agriculture (Rubio et al., 2014). The present study focuses on the role a *T. parareesei* CM in biocontrol and the induction of plant responses. This enzyme catalyzes the conversion of chorismate to prephenate, the precursor of the AAA Tyr and Phe, and constitutes a diverted node of the shikimate pathway, a biosynthetic network for primary and secondary metabolites. Contrary to what has been reported for *T. reesei*, with limited biocontrol activity, *T. parareesei* is able to antagonize fungal and oomycete plant pathogens (Atanasova et al., 2010; Rubio et al., 2014). Since the genome of *T. parareesei* has not been annotated and no functional studies of the shikimate pathway have been reported in *Trichoderma*, we applied a genomic approach, taking advantage of the whole genome sequence of *T. reesei* (Martinez et al., 2008). At least eight genes encoding putative enzymes of the shikimate pathway from E-4P and PEP to prephenate were identified in *T. reesei* (Figure 1), and the one corresponding to a CM was used to design oligonucleotides to isolate the *Tparo7* gene in *T. parareesei*. As expected, among the CMs previously characterized functionally TpARO7 showed the highest identity with the cytosolic CMs of filamentous ascomycetes (Krappmann et al., 1999). In addition, TpARO7 displayed more sequence identity with CMs from bacteria and plants than that observed with the secreted Cmu1 of *U. maydis* (Djamei et al., 2011). The catalytic residues R^20^, E^27^, R^162^, K^173^, T^250^, and E^254^, which have been shown to be essential for the activity of some previously reported CMs, were also present in TpARO7.

CMs are normally feedback-inhibited by Tyr and/or Phe and induced by Trp, indicating allosteric regulation by AAA (Krappmann et al., 1999; Tzin and Galili, 2010). The *in vitro* CM activities calculated after the incubation of *T. parareesei* T6 protein extracts in the presence of AAA revealed feedback inhibition by Tyr and induction by Trp. Although *Tparo7* transcript levels were significantly upregulated in strain T6 grown in MM supplemented with chorismic acid or AAA (Figure S1), the corresponding CM activities in intracellular protein from mycelia cultured in these media were lower than those calculated in MM (Table 1). These results are compatible with the above-indicated regulation of CM by allosteric feedback-control of the different AAA. Since saprotrophic fungal growth has to adapt according to the surrounding environmental conditions, when AAA are scarce their biosynthesis should be regulated. Perhaps this occurred when T6 was incubated under imbalanced AAA sources, since many fungi possess the cross-pathway control system required to overcome an imbalanced amino acids diet, which is a complex process due to several feedback- or cross-feedback-controlled multi-amino acid pathways (Singh et al., 2010).

We generated CM mutant strains by RNA-mediated *Tparo7* gene silencing. This strategy allows the functions of genes to be studied under conditions where they are essential, since small transcript levels are sufficient to keep the organism viable. This approach proved to be adequate to analyze genes functionally in *T. harzianum* (Cardoza et al., 2006). As in previous studies performed with *Trichoderma* silenced transformants (Cardoza et al., 2006; Morán-Diez et al., 2009), different degrees of silencing were observed among the *Tparo7*-silenced mutants at both the transcript and CM activity levels (Figure 2 and Table 2). We observed that *Tparo7* expression and CM activity were related, and only a residual enzymatic activity was detected for the silenced transformant Tparo7-S4, which showed the lowest transcript levels. No correlation was observed between the number of pSIL-ARO7 copies inserted and the gene expression levels and CM activity. In fact, compared to the T6 and the Tp-TC strains, the highest *Tparo7* expression observed in Tparo7-S2 grown in PDB (Figure S2) was accompanied by the highest CM activity. This same lack of correlation has also been reported for some other genes overexpressed or silenced in *Trichoderma* (Limón et al., 1999; Montero-Barrientos et al., 2007; Morán-Diez et al., 2009). The differences in *Tparo7* expression and CM activity between T6 and Tp-TC observed in some culture media are indicative of the fact that the insertion of the transformation cassette can affect this key regulation point of the primary metabolism under such culture conditions. For this reason, both strains were included in all tests carried out in the present study, displaying similar behavior in most of them.

As expected for a gene encoding a primary metabolism major node enzyme, *Tparo7*-silenced strains showed lower growth rates than those of T6 or Tp-TC on different culture media (Table 3). This phenotype was more evident in MM, mainly when this medium was supplemented with a mixture of AAA. This suggests an allosteric feedback-control of TpARO7 by AAA. Interestingly, Tparo7-S4, the strain with the lowest CM activity, showed the lowest growth rates at early incubation times (see Figure S3). However, this silenced mutant presented faster growth than Tparo7-S3 between 24 and 48 h of incubation (Table 3). This behavior, observed in several media, could be provoked by the complex regulation of the shikimate pathway just to stay alive despite of its high degree of CM silencing.

It is known that the biocontrol potential of *Trichoderma* varies depending upon the strains confronted (Atanasova et al., 2013). It could be proposed that the lower growth rate of Tparo-S3 and Tparo-S4 could be the cause of the reduced antagonistic ability displayed by these mutants against FO, RS and BC in dual cultures (Figure 3 and Figure S3). However, the limited mycoparasitic efficiency of the silenced mutants against FO and BC cannot be explained on the basis of fitness or substrate competition, but instead by other *Trichoderma* biocontrol mechanisms. *Tparo7* silencing affected CW-degrading enzyme production in terms of reduced protease and increased chitinase activities. Recent studies based on transcriptomic analyses have indicated that proteolysis is a major biological process involved in the mycoparasitism of *Trichoderma* overgrowing its prey (Atanasova et al., 2013; Steindorff et al., 2014). The increased chitinase activity detected in *Tparo7*-silenced strains has also been reported when the *tri4* gene, involved in trichothecene production, was disrupted or silenced in *T. arundinaceum* (Malmierca et al., 2012). It has been documented that exogenous supply of SA or culture media, which contain high levels of SA, induce chitinase activity in plant cells (Schneider-Müller et al., 1994), and that Ca^2+^ plays an important role in the production of chitinase and SA (Schneider-Müller et al., 1994; Kawano et al., 1998). Although Ca^2+^ concentrations were not calculated in the present study, we have observed a correlation between SA production and chitinase activity in the different *T. parareesei* strains. In particular, Tparo7-S3 showed the maximum SA levels accompanied by the highest chitinase activity. In any case, this correlation needs to be further explored in fungi. These results would justify the unexpected high BC inhibition activity detected in membrane assays for Tparo7-S3. In these assays, performed on medium where *T. parareesei* was previously grown for 24 h, the above indicated scarce growth of Tparo7-S4 during the first hours of incubation could explain its low inhibition percentages recorded against the three pathogens (Table 4). However, membrane and supernatant assays have different experimental designs and results cannot be compared. In the tests performed with 48 h-PDB supernatants, those from Tparo7-S3 showed the highest antifungal activity against FO and BC, the two pathogens assayed (Figure 4). Moreover, taking into account that Tparo7-S4 has less growth than T6 and Tp-TC, its supernatant did not show reduced antifungal activity against these two pathogens at different incubation times. This is in agreement with the increased production of the AAA-derived antifungal compounds 2-PE, tyrosol and SA detected in both *Tparo7*-silenced mutants (Table 6). Thus, *Tparo7* silencing has a positive effect on metabolite production in *T. parareesei*. An explanation for this is that CM activity could be compromised in *Tparo7*-silenced mutants and it has been reported that this enzyme is allosterically regulated by Tyr and Phe (Krappmann et al., 1999; Tzin and Galili, 2010). Thus, the conversion of Tyr and Phe into tyrosol and 2-PE respectively would prevent the allosteric inhibition of CM by these two amino acids. The production of tyrosol and 2-PE has been reported previously in *Trichoderma* spp. (Tarus et al., 2003), and a collateral tyrosol production has been identified in *T. brevicompactum* mutants overexpressing a trichodiene synthase involved in the biosynthesis of trichothecene compounds with antifungal activity (Tijerino et al., 2011). It has also been described previously that tyrosol, 2-PE and SA are part of the biocontrol strategies of yeast and bacteria against phytopathogens (Mercado-Blanco et al., 2001; Tarus et al., 2003; Liu et al., 2014). Bearing in mind the biocontrol assay data, the SM levels resulting from *Tparo7* silencing would affect the production of CW-degrading enzymes and hence the mycoparasitic activity of *T. parareesei*.

Successful colonization is considered to be a major premise for the beneficial effects exerted by *Trichoderma* on plants (Harman et al., 2004; Morán-Diez et al., 2009; Hermosa et al., 2012). Our results concerning tomato root colonization by *T. parareesei* strains are in agreement with the growth rates calculated in different media. However, if the lower root colonization capacity observed for *Tparo7*-silenced mutants as compared to those of the control strains (Table 7) were a direct consequence of their limited growth, there would be insufficient data to support the notion that TpARO7 plays a major role in the *T. parareesei* rhizosphere colonization process. Since several reports have shown that *Trichoderma* SMs can affect plant growth and defense (Viterbo et al., 2007; Vinale et al., 2008; Tijerino et al., 2011; Malmierca et al., 2012, 2015), we used *in vivo* assays to explore the role of *Tparo7* in *T. parareesei*-tomato interactions. In comparison with control plants, we observed significantly lower growth parameters in plants whose seeds had been coated with *Tparo7*-silenced mutants (Table 8); this could be explained by their higher SA production (Table 6). We also observed that exogenous applications of 2–100 μM tyrosol increased tomato seed germination but concentrations higher than 250 μM elicited the opposite effect (data not shown). It has been reported that the effect of exogenous SA on growth depends on the plant species, the developmental stage and the SA concentrations tested, since low amounts of SA can have growth-stimulating effects but higher SA concentrations have been associated with reduced growth and chlorophyll content (Kovácik et al., 2009; Rivas-San Vicente and Plasencia, 2011). We analyzed the production of tyrosol, 2-PE and SA by *T. parareesei* strains, but differences in the biosynthesis of other metabolites could occur after *Tparo7* silencing, since the shikimate pathway is strongly regulated and a huge variety of metabolites can be derived from it. We also observed that tomato plants treated with *Tparo7*-silenced mutants showed reduced protection against BC, which would be in agreement with the observed downregulation of *LOX1* and *EIN2* (Figure 5), involved in induced systemic resistance against necrotrophs.

As far as we know this is the first report relating CM silencing with biocontrol. The results concerning CM reported in this article support the hypothesis that SMs produced in the shikimate pathway are involved in the biocontrol potential of *T. parareesei* and some of them appear to be key molecules for maintaining a proper balance in the responses elicited by *T. parareesei* during its interaction with plants. The observed relationships between AAA-derived SM production and CW degrading enzyme activity, already described in plants, need to be explained in fungi and the *T. parareesei* CM-silenced mutants obtained in the present work can be a touchstone to pursuing this goal.

### Conflict of interest statement

The authors declare that the research was conducted in the absence of any commercial or financial relationships that could be construed as a potential conflict of interest.

## Supplementary material

Figure S1 **Expression of *Tparo7* gene in *T. parareesei* T6 by real-time PCR**. Total RNA was extracted from mycelia grown for 48 h on PDB and transferred to MM containing 2% glucose (MM) or 5 mM Tyr (MM-Tyr), Phe (MM-Phe), Trp (MM-Trp) or chorismic acid (MM-chorismic acid) for 24 h. Values correspond to relative measurements against the *Tparo7* transcript in T6 grown in MM containing 2% glucose (2^−ΔΔCt^ = 1). *T. parareesei* T6 actin was used as internal reference gene. Bars represent the standard deviations of the mean of three replicates. Asterisk (^*^) represents statistically significant differences (*P* < 0.05).

Figure S2 **Southern blot analysis of wild type (T6) and transformant strains**. Genomic DNAs were *SacI*-digested and the *phleomycin* gene was used as probe. *T. parareesei* T6 (line 1), Tparo7-S1 (line 2), Tparo7-S2 (line 3), Tparo7-S3 (line 4), and Tparo7-S4 (line 5). *HindIII*-digested λ DNA was used as a marker and molecular sizes are indicated in kbp (line M).

Figure S3 **Dual cultures of strains T6, the silenced transformants Tparo7-S3 and Tparo7-S4, and the control transformant Tp-TC of *T. parareesei* and the pathogen *B. cinerea* (BC) on PDA medium**. Plates in the center correspond to the pathogen growth without *Trichoderma* strain. Plates were incubated at 28°C for 18 (**A**), 48 (**B**), and 72 (**C**) h.

Figure S4 **Necrotic lesions observed in tomato leaves after *T. parareesei* seed treatment and *B. cinerea* conidia infection**. Untreated seed and *B. cinerea*-infected leaves (control) and *T. parareesei* T6-, control transformant Tp-TC-, silenced transformants Tparo7-S3- and Tparo7-S4-treated seed and B. cinerea-infected leaves. Image was taken three days after *B. cinerea*-infection.
