# Supplementary figures and images for: The importance of chorismate mutase in the biocontrol potential of Trichoderma parareesei
Source: Front Microbiol. 2015 Oct 27;6:1181. doi: 10.3389/fmicb.2015.01181 (PMC4621298; doi:10.3389/fmicb.2015.01181)

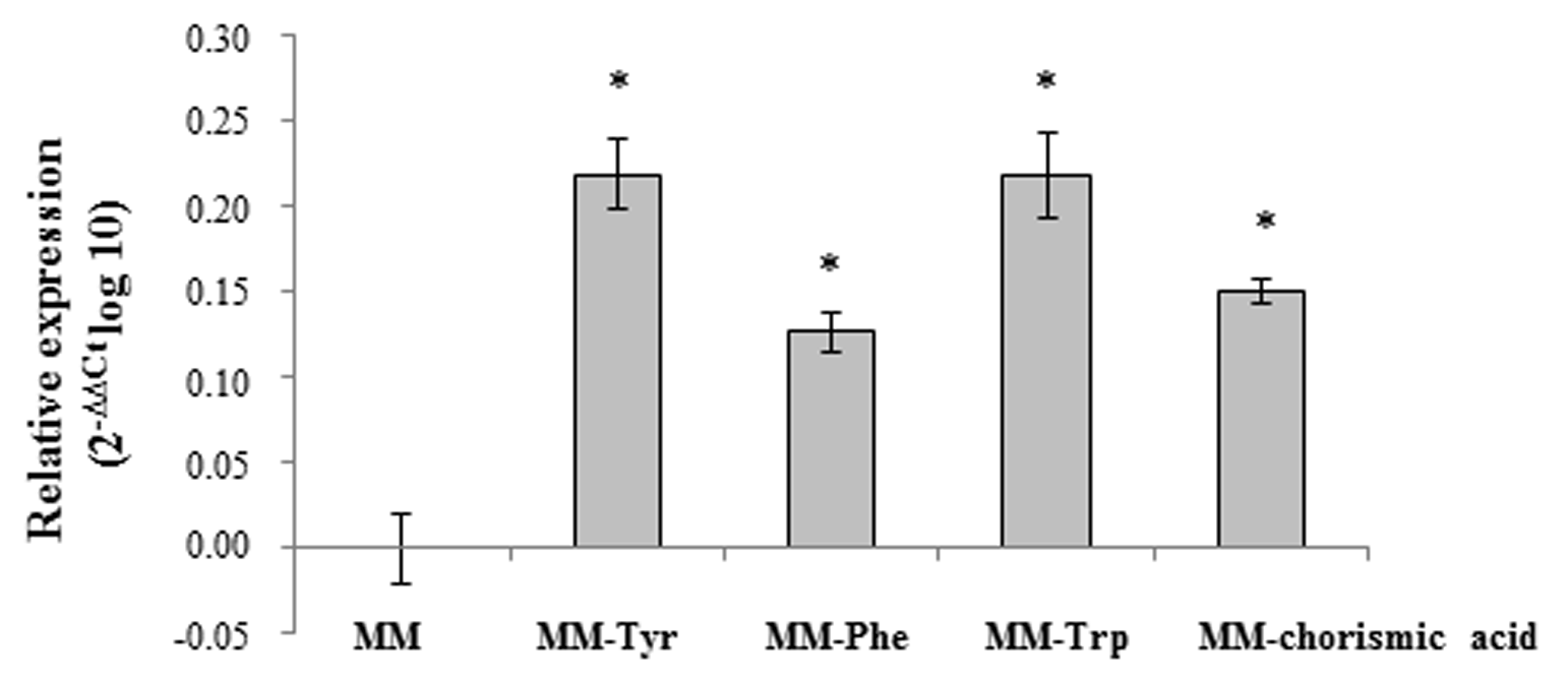

Supplement: Figure S1 — Expression of Tparo7 gene in T. parareesei T6 by real-time PCR. Total RNA was extracted from mycelia grown for 48 h on PDB and transferred to MM containing 2% glucose (MM) or 5 mM Tyr (MM-Tyr), Phe (MM-Phe), Trp (MM-Trp) or chorismic acid (MM-chorismic acid) for 24 h. Values correspond to relative measurements against the Tparo7 transcript in T6 grown in MM containing 2% glucose (2−ΔΔCt = 1). T. parareesei T6 actin was used as internal reference gene. Bars represent the standard deviations of the mean of three replicates. Asterisk (*) represents statistically significant differences (P < 0.05). [file Image1.TIFF]

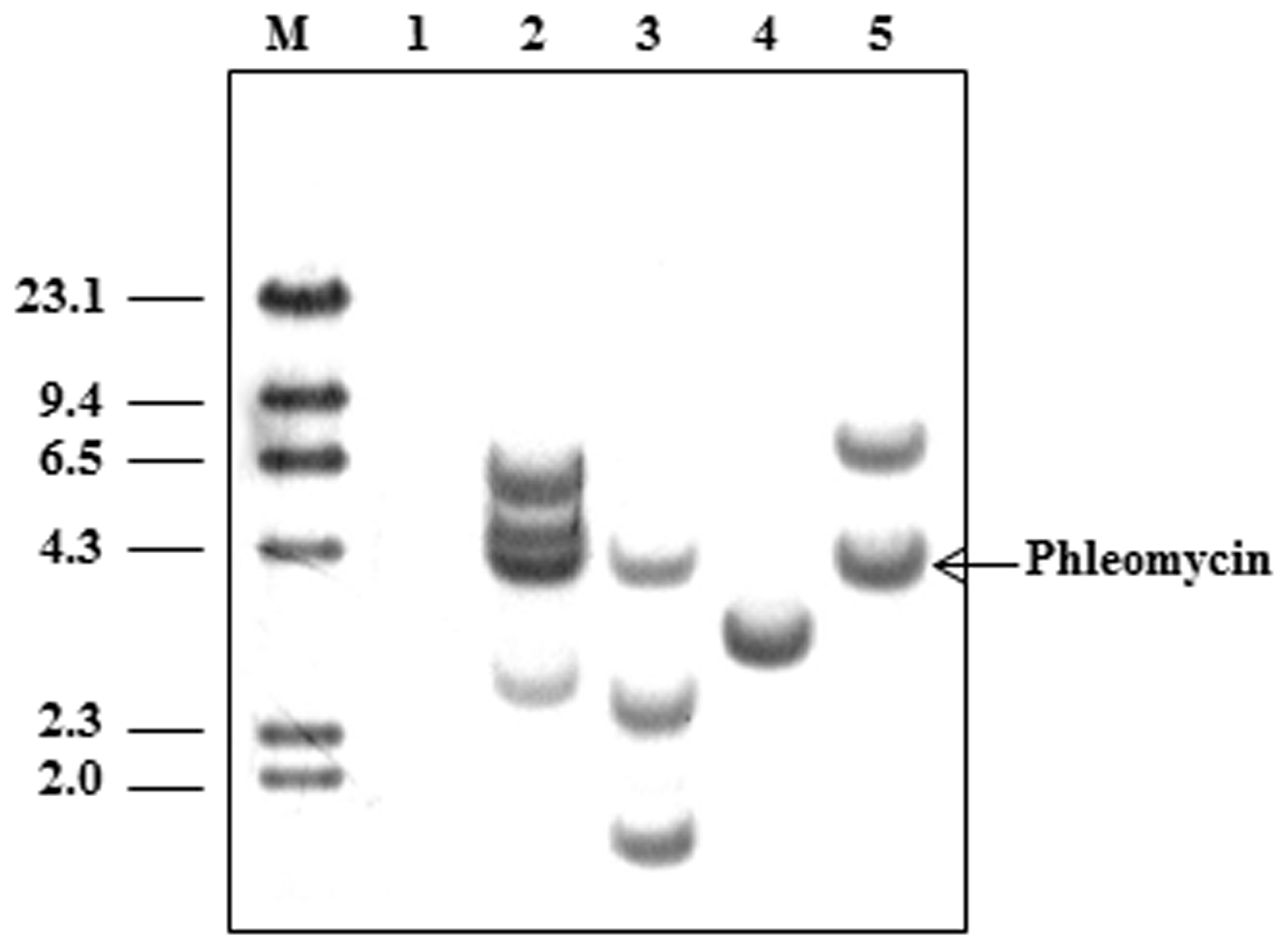

Supplement: Figure S2 — Southern blot analysis of wild type (T6) and transformant strains. Genomic DNAs were SacI-digested and the phleomycin gene was used as probe. T. parareesei T6 (line 1), Tparo7-S1 (line 2), Tparo7-S2 (line 3), Tparo7-S3 (line 4), and Tparo7-S4 (line 5). HindIII-digested λ DNA was used as a marker and molecular sizes are indicated in kbp (line M). [file Image2.TIFF]

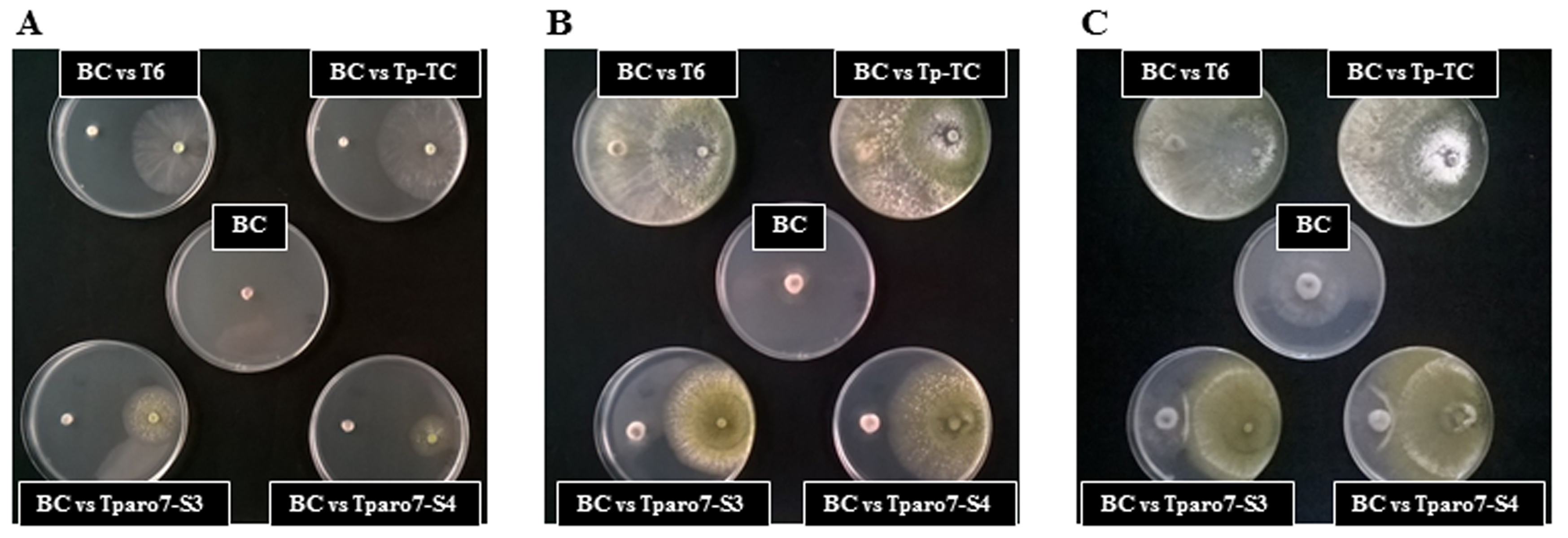

Supplement: Figure S3 — Dual cultures of strains T6, the silenced transformants Tparo7-S3 and Tparo7-S4, and the control transformant Tp-TC of T. parareesei and the pathogen B. cinerea (BC) on PDA medium. Plates in the center correspond to the pathogen growth without Trichoderma strain. Plates were incubated at 28°C for 18 (A), 48 (B), and 72 (C) h. [file Image3.TIFF]

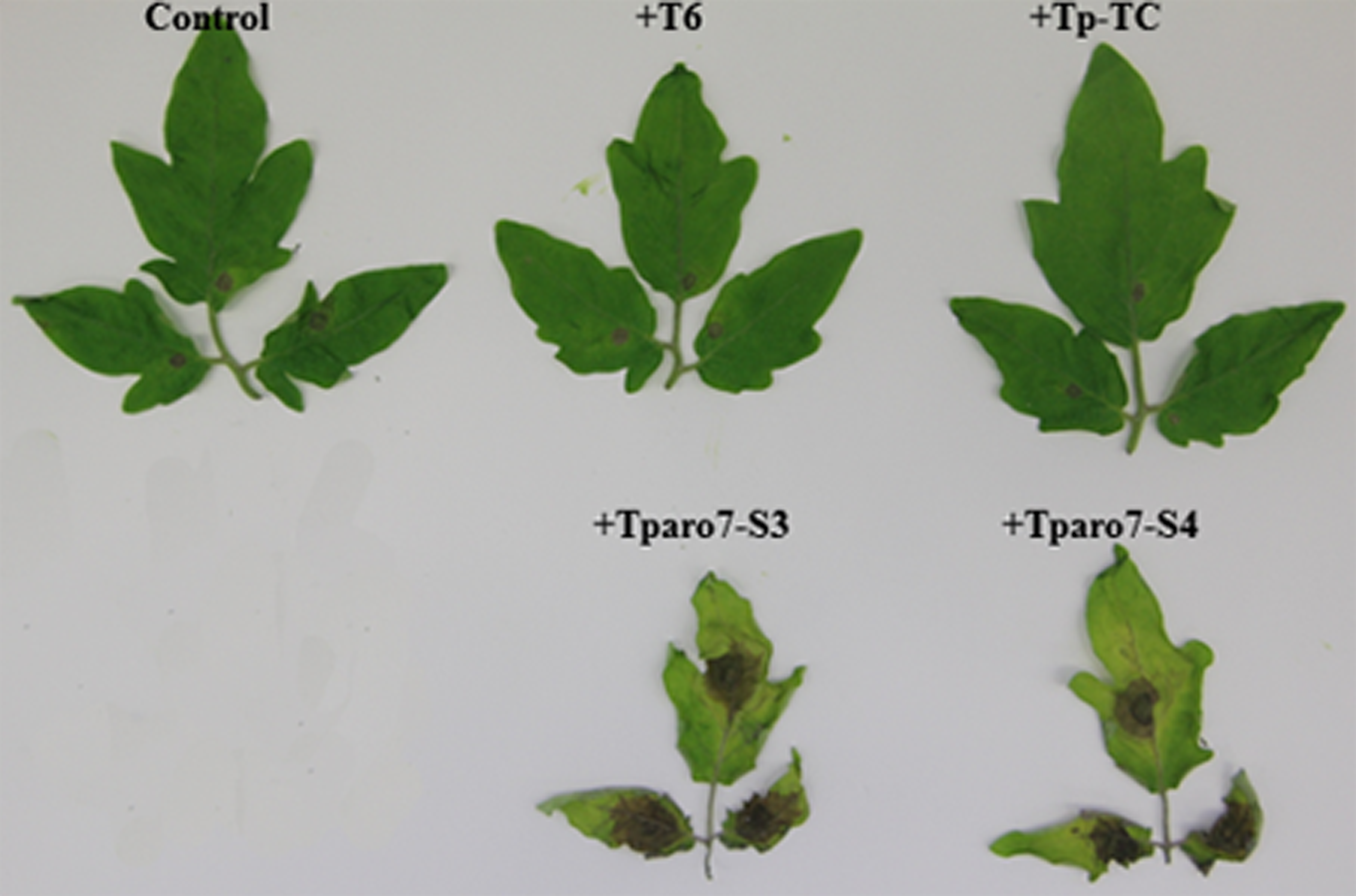

Supplement: Figure S4 — Necrotic lesions observed in tomato leaves after T. parareesei seed treatment and B. cinerea conidia infection. Untreated seed and B. cinerea-infected leaves (control) and T. parareesei T6-, control transformant Tp-TC-, silenced transformants Tparo7-S3- and Tparo7-S4-treated seed and B. cinerea-infected leaves. Image was taken three days after B. cinerea-infection. [file Image4.TIFF]
