# Supplementary material for: The importance of chorismate mutase in the biocontrol potential of Trichoderma parareesei
Source: Front Microbiol. 2015 Oct 27;6:1181. doi: 10.3389/fmicb.2015.01181 (PMC4621298; doi:10.3389/fmicb.2015.01181)
Supplement: Supplementary file 5 [file Table1.DOCX]

**TABLE S1. Primers used in the present study.**

| **Primer** | **Target gene** | **Sequence (5’-3’)** | **Slope** | **Efficiency** |
| --- | --- | --- | --- | --- |
| **For gene isolation** |  |  |  |  |
| Tparo7-F | *Tparo7* | ATGGATTCAGCCGTCGACATG |  |  |
| Tparo7-R |  | CTACTCTTCGGGTATGAGCC |  |  |
| **For construction of silencing plasmid** |  |  |  |  |
| Aro7-SpeI | *Tparo7* | ACTAGTGCAATCTCCATCCC |  |  |
| Aro7-BamHI |  | GGATCCTACGTCTCGACCTT |  |  |
| Aro7-XhoI |  | CTCGAGGCAATCTCCATCCC |  |  |
| Aro7-HindIII |  | AAGCTTTACGTCTCGACCTT |  |  |
| **For screening of transformant strains** |  |  |  |  |
| TADIR2 |  | TGACCACTTCGCTGCCTATC |  |  |
| Intro-R |  | CATCCATGATAAGAGTCTGAG |  |  |
| **For qRT-PCR** |  |  |  |  |
| Qaro7-C | *Tparo7* | CGTCCACCTGCGACATTG | -3.28 | 101.83 |
| Qaro7-D |  | GCCTCTGCATCAAGTACTC |  |  |
| Act-1-tricho | *actin* | ATCGGTATGGGTCAGAAGGA | -3.12 | 108.94 |
| Act-2-tricho |  | ATGTCAACACGAGCAATGG |  |  |
| Act-F | *actin* | ATCGGTATGGGTCAGAAGGA | -3.35 | 98.89 |
| Act-R |  | ATGTCAACACGAGCAATGG |  |  |
| Ein2-fw | *EIN2* | GTTGCTAAGTGATGCTGTA | -3.46 | 94.54 |
| Ein2-rev |  | CGCTCAAGCATGCTGGGCC |  |  |
| Lox1-fw | *LOX1* | GCCTCTCTTCTTGATGGAG | -3.23 | 103.56 |
| Lox1-rev |  | GTAGTGAGCCACTTCTCCAA |  |  |
| PR1-fw | *PR-1* | CCTCAAGATTATCTTAACGCTC | -3.51 | 92.67 |
| PR1-rev |  | TACCATTGCTTCTCATCAACC |  |  |
