# Supplementary material for: The importance of chorismate mutase in the biocontrol potential of Trichoderma parareesei
Source: Front Microbiol. 2015 Oct 27;6:1181. doi: 10.3389/fmicb.2015.01181 (PMC4621298; doi:10.3389/fmicb.2015.01181)
Supplement: Supplementary file 6 [file Table2.DOC]

**TABLE S2.** Effect of tyrosol, salicylic acid and 2-phenylethanol on the inhibition (%) of mycelial growth of *F. oxysporum* and *R. solani* compared to PDA medium. In each column and for each product, means followed by different superscript letters are significantly different (*P* < 0.05).

|  | | ***F. oxysporum*** | ***R. solani*** |
| --- | --- | --- | --- |
| **Tyrosol** | **5 mM** | 22.3 ^c^ | 7.7 ^c^ |
|  | **10 mM** | 37.9 ^b^ | 20.5 ^b^ |
|  | **30 mM** | 56.9 ^a^ | 58.5 ^a^ |
| **Salicylic Acid** | **5 mM** | 50.4 ^c^ | 58.3 ^b^ |
|  | **10 mM** | 61.8 ^b^ | 100.0 ^a^ |
|  | **30 mM** | 100.0 ^a^ | 100.0 ^a^ |
| **2-Phenylethanol** | **5 mM** | 14.3 ^c^ | 43.4 ^c^ |
|  | **10 mM** | 55.2 ^b^ | 79.2 ^b^ |
|  | **30 mM** | 100.0 ^a^ | 100.0 ^a^ |
|  |  |  |  |
